# Citizen engagement in healthcare procurement decision-making by healthcare insurers: recent experiences in the Netherlands

**DOI:** 10.1186/s12961-022-00939-7

**Published:** 2022-12-22

**Authors:** Óscar Brito Fernandes, Véronique Bos, Niek Klazinga, Dionne Kringos

**Affiliations:** 1 Department of Health Economics, Corvinus University of Budapest, Fővám tér 8, Budapest, 1093 Hungary; 2 Amsterdam UMC location University of Amsterdam, Public and Occupational Health, Meibergdreef 9, Amsterdam, The Netherlands; 3 Public Health research institute, Quality of Care, Amsterdam, The Netherlands

**Keywords:** Managed competition, Social health insurance, Citizen engagement, Purchasing, People-centred healthcare, Value-based healthcare

## Abstract

**Background:**

In insurance-based healthcare systems, healthcare insurers are interested in engaging citizens in care procurement to contract healthcare services that matter to people. In the Netherlands, an amendment to the Health Insurance Act was set forth in 2021 to formalize and strengthen the engagement of the insured population with healthcare insurers’ procurement cycles. This study explores the role of Dutch healthcare insurers in operationalizing citizen engagement in procurement cycles before changes occur linked to the amendment to the Health Insurance Act.

**Methods:**

A phenomenological qualitative design was employed in two phases: (1) we consulted academics and policy experts on the role of healthcare insurers regarding citizen engagement; (2) we conducted focus groups with representatives of healthcare insurers to understand how citizens’ engagement is being operationalized. Transcripts of the interviews with experts and detailed notes of focus group meetings were analysed using a qualitative inductive approach. Selected excerpts were analysed on discourse and content and organized by a coding scheme following a rigorous and accelerated data reduction technique.

**Results:**

We identified four strategies used by healthcare insurers to operationalize citizen engagement: (1) broadening their population health orientation; (2) developing and improving mechanisms for engaging citizens; (3) strengthening features of data governance for effective use of value-driven data; (4) implementing financial and incentive mechanisms among healthcare providers in support of value-based healthcare. However, regulated market mechanisms and low institutional trust in healthcare insurers undermine their transition from merely funding healthcare towards becoming people-centred value-based healthcare purchasers.

**Conclusion:**

Dutch healthcare insurers seem to be strengthening the community orientation of their functioning while enhancing the end-to-end experience of the insured. The expected practical effects of the amendment to the Health Insurance Act include broadening the role of the council of insurees in decision-making processes and systematically documenting the efforts set forth by healthcare insurers in engaging citizens. Further research is needed to better understand how the regulated competitive market could be hampering the engagement of citizens in healthcare procurement decision-making and value creation from the citizens’ perspective.

**Supplementary Information:**

The online version contains supplementary material available at 10.1186/s12961-022-00939-7.

## Background

To achieve the goals of providing care and resources to foster good health among populations, healthcare systems are increasingly engaging citizens in co-designing the system [1–3]. The increased openness of healthcare systems to citizens’ voice confers the public greater power in decision-making processes and can help hold systems and actors accountable. Engaging citizens has been supported by notions of people-centredness in healthcare systems [1, 2] and later their translation to value-based approaches to care to improve people’s care experiences, improve the health of populations and reduce per capita costs of care for populations (triple aim) [4, 5]. A people-centred approach to care adopts individuals’, carers’, families’ and communities’ perspectives as participants in and beneficiaries of healthcare systems organized around the comprehensive needs, preferences and values of people rather than diseases. By becoming more people-centred, healthcare systems have been seeking to embed citizens’ voice in decision-making cycles and strengthening citizens’ agency in shaping the healthcare system, notably by measuring outcomes and experiences of care from the perspective of healthcare service users and patients.

Terms such as citizen, patient, customer/client and insured encompass different roles; these roles shape both relationships and behaviours [6]. In their broader participation in society, citizens are also healthcare service users or patients. Citizens can also assume the role of customers/clients whose interests are protected by an insurance policy (insuree). Although differentiating these roles may seem artificial, clarifying this is relevant in health policy decision-making cycles to ensure that the proper perspectives are considered.

In insurance-based healthcare systems, procurement is a core function of healthcare insurers’ business models. Procurement practices have evolved in tandem with healthcare systems becoming responsive to citizens’ needs, expectations and preferences [7]. Hence, embedding citizens’ voice has become a crucial requirement for fostering value-based procurement in people-centred healthcare systems [8]. However, value creation through negotiating price, volume, care quality and outcomes is demanding and time-consuming for healthcare insurers [9–11].

Healthcare insurers have a unique position to serve patients in their healthcare needs and support the broader population in accessing people-centred value-based healthcare services. In fulfilling their function of procuring people-centred value-based healthcare services, healthcare insurers are developing their data strategies to gain insights into value creation from the citizens’ perspective, notably by collecting people-reported data [12]. Yet, there is a chasm between the data healthcare insurers collect from the insured and how data are used towards realizing people-centred value-based healthcare, as highlighted by a scoping review conducted by members of this research group [12].

Healthcare insurance in the Netherlands has its roots in solidarity values. It developed from the financial assistance from guild “collecting-boxes” for specific groups in the seventeenth century to a social insurance model with Sick Funds (adopted in 1941) for part of the population under a certain income level; and, more recently, towards a population-wide social health insurance model since the introduction of the Health Insurance Act in 2006 [13–15]. The Health Insurance Act steered the functioning of financing and procurement of medical care to be organized and governed via three regulated competitive markets: (1) a healthcare insurance market between healthcare insurers and the insured; (2) a healthcare purchasing market between healthcare insurers and care providers; (3) a healthcare provision market, between care providers and citizens [11, 16]. The regulated markets should reflect the societal values regarded as worthy, such as solidarity, rooted in an underlying risk-sharing approach from the time of sickness funds.

Currently, healthcare insurers are focusing on other aspects of care beyond cost containment and cost-effectiveness. They are also involved in ensuring the adequacy of care delivery and planning for improved health of the (insured) population, thus becoming a proactive purchaser of care quality and person-centred care [16]. In 2021, an amendment to the Health Insurance Act was introduced to strengthen the influence of the insured on healthcare insurers (*wet verzekerdeninvloed*) [17]. The amendment’s goal was to foster opportunities for insurees to express their views about procurement policies in support of a people-centred value-based healthcare system.

Citizen engagement is key in a regulated competitive market to steer actors towards realizing a people-centred value-based healthcare system [18]. Healthcare insurers have been operationalizing several applications of citizen engagement (e.g. by conducting surveys and focus groups) and using intelligence derived from these applications for ensuring and improving care quality and bolstering the value-based healthcare agenda [12]. However, the extent to which these applications of citizen engagement serve a tokenistic function in the relationship between healthcare insurers and citizens (including insurees) remains overlooked. We aim to explore the role of healthcare insurers in operationalizing citizen engagement in healthcare procurement decision-making in the Dutch healthcare system. Notably, this study sheds light on how the rollout of an amendment to the Health Insurance Act is expected to change healthcare insurers’ practices of engaging citizens in healthcare procurement decision-making processes. We formulated two research questions: (1) How is citizen engagement by healthcare insurers embedded in the Dutch healthcare system? (2) How do healthcare insurers perceive their role concerning citizen engagement, and how do they operationalize this role?

## Methods

### Design

This study is situated in the interpretivism paradigm; it followed a phenomenological qualitative design employed in two phases (Table 1). In phase 1, we consulted academics and policy experts to create an essential understanding of the role of healthcare insurers in the Dutch context. From these interviews, we drew an external perspective of the role of healthcare insurers in the Dutch healthcare system. Relevant context developments signalled by the experts were translated into discussion statements to be debated with representatives of healthcare insurers. In phase 2, six focus groups were convened with a purposeful sample of representatives of the four largest Dutch healthcare insurers. During focus group meetings, healthcare insurers reflected on the developments in the Dutch context, resulting in an internal perspective of their role in the Dutch healthcare system. The focus groups were held virtually to accommodate public health restrictions during the COVID-19 pandemic. According to Dutch law, the study was exempt from formal ethics approval since it did not involve subjecting participants to medical interventions. All participants were informed beforehand about privacy and data-security aspects and participated voluntarily.

**Table 1:** Overview of the timeline and research processes

	Phase 1	Phase 2
Participants	Academics and policy experts	Representatives of healthcare insurers
Period	December 2019 to January 2020	October 2020 to March 2021
Aim	To create an essential understanding of the role of healthcare insurers in the Dutch context	To explore the perceptions of healthcare insurers on their role with respect to citizen engagement and the operationalization thereof
Method	In-person and at-distance semi-structured interviews	Online focus groups
Steps	1.1. Selecting participants based on criteria (e.g. authorship of scientific literature on the topic of interest) 1.2. Conducting interviews with experts (*n* = 11) 1.3. Analysing interview materials 1.4. Drawing key discussion statements to be debated with representatives of healthcare insurers in phase 2	2.1. Pitching the study to a person of interest at the healthcare insurer 2.2. Identifying a focal point at the healthcare insurer 2.3. Reasoning with the focal point on the profiles of intended focus group participants 2.4. In-house recruiting of participants conducted by the focal point (*n* = 29) 2.5. Sending the invitation letter and materials to participants 2.6. Hosting the focus group meeting (*n* = 6) 2.7. Preparing a key summary of the meeting and reverting to participants for feedback 2.8. Following-up on the feedback of participants 2.9. Analysing focus group materials

### Selection of participants

Considering the aim of the study and its phenomenological qualitative design, we aimed at learning from first-hand experiences of key actors, notably academics and policy experts, and representatives of healthcare insurers. These two perspectives—the former emphasizing research and policy considerations, and the latter providing more in-depth knowledge about healthcare insurers’ business models—allowed a broader understanding of the market forces in the Dutch healthcare system and healthcare insurers’ processes regarding healthcare procurement and the extent to which citizens are included in these processes.

#### Academics and policy experts

We drafted a list of academics and policy experts using the following criteria: (1) the authorship of scientific literature retrieved via a quick literature scan; (2) past working experience; (3) referrals via the network of the research group. Our goal was to contact experts with a research or policy background in healthcare insurance in the Netherlands. The aim was to interview 10–15 experts (Additional file 1).

#### Representatives of healthcare insurers

As of 2021, Dutch healthcare insurers were consolidated into 23 competing insurers operating under 11 health insurance companies. We chose to contact the four largest health insurance companies (VGZ, Achmea, CZ and Menzis) for two reasons: (1) to reach a broad representation of the health insurance market, considering that these four companies hold 81% of the market share, and (2) to understand the extent to which the consolidation of the regulated competitive market (e.g. the merging of healthcare insurers over the years into larger corporate firms) has still made it possible for these companies to connect with citizens’ needs and expectations, notably, to understand changes to the historical background rooted in the solidarity values of many healthcare insurers.

For each healthcare insurer, a focal point was identified. We reasoned with each focal point on the profiles of prospective focus group participants (people with a role in realizing citizen engagement at the healthcare insurer, particularly those with a role in implementing the amendment to the Health Insurance Act). After that, the focal point was responsible for the in-house recruitment of participants. Before the focus group meeting, each participant received an invitation letter from the research team detailing the scope, background, aim and general guiding themes to be explored during the session (Additional file 2). The aim was to conduct at least one focus group per healthcare insurer and to engage approximately 20 participants.

### Data collection

#### Semi-structured interviews with academics and policy experts

We conducted explorative semi-structured interviews with 11 experts between December 2019 and January 2020. Interviews were conducted in English, lasted on average 45 minutes, and addressed three main themes: (1) the role of healthcare insurers in the Dutch healthcare system; (2) the developments in and operationalization of citizen engagement by healthcare insurers; (3) the uses of people- and patient-reported data by healthcare insurers. One researcher (ÓBF) conducted interviews, which were audio-recorded and transcribed verbatim. The interviews were conducted both in person and by phone or videoconferencing based on the proximity and preference of experts.

#### Focus groups with representatives of healthcare insurers

We conducted six focus groups with representatives of healthcare insurers between October 2020 and March 2021. Each focus group meeting lasted, on average, 90 minutes. The focus groups were held in English and Dutch to allow participants to communicate in the language that best suited them. The meeting was structured in two parts. First, to contextualize the study, ÓBF shared conclusions of the scoping review performed earlier by members of this research group on the use of patient-reported data by healthcare insurers [12]. Secondly, NK moderated the discussion around four discussion statements drawn from phase 1 of the study (these were displayed in both English and Dutch) while ÓBF, VB, and DK took notes (Additional file 3). The video recording and detailed meeting notes were reviewed in parallel to produce key messages. Participants then had the chance to review and validate these key messages regarding the accuracy, missed points and last thoughts.

### Data analysis

The raw data consisted of transcribed interviews with experts and video recordings and notes of focus group meetings. Two researchers (ÓBF and VB) acquainted themselves with all the raw data. Transcripts of the interviews with experts were analysed using a qualitative inductive approach. One researcher (ÓBF) extracted excerpts from the transcripts and stored them in a spreadsheet. The excerpts were analysed on discourse and content and organized by a coding scheme following a rigorous and accelerated data reduction technique [19]. Before the systematic text consolidation, emerging themes were discussed iteratively among the research team. A second researcher (VB) reviewed the analysis of the excerpts. Potential disagreements in coding were checked with the raw data. A similar approach was followed with the data produced during the focus group meetings.

## Results

In total, 40 people were engaged during the two phases of the study: 11 experts in phase 1 and 29 focus group participants in phase 2 (Table 2).

**Table 2:** Characteristics of the participants by study phase

Phase 1		Phase 2	
Characteristics of academics and policy experts		Characteristics of representatives of healthcare insurers	
Role	*n* = 11	Role	*n* = 29
Policy-maker	2	Council of insurees	5
Provider	1	Management	10
Researcher	7	Marketing and sales	4
Strategy consultant	1	Policy advisor	10
Sex		Sex	
Female	3	Female	21
Male	8	Male	8

### The role of healthcare insurers in citizen engagement in the Dutch healthcare system

The experts recognized that healthcare insurers, via competition mechanisms of the regulated market, serve insured citizens’ interests (e.g. ensuring the affordability of health insurance premiums). However, some experts noted tensions in the healthcare purchasing and insurance markets because of a misalignment between the expectations of the role of healthcare insurers and that observed/experienced by other actors in the healthcare system, such as care providers and citizens. For example, while citizens expect healthcare insurers to be more involved with health promotion and preventive care, these functions are not yet fully embedded in healthcare insurers’ business models (Expert_6.70, Additional file 4).

Some experts signalled that the amendment to the Health Insurance Act (*wet verzekerdeninvloed*) builds off public pressure on healthcare insurers as part of a broader discussion about the benefits of the regulated market. Two key aspects underpin these discussions. The first is how to make healthcare insurers accountable for the engagement of their insured. Most of the experts noted that healthcare insurers had already been engaging their insured in decision-making (and, to some extent, citizens in general) before the amendment to the law, but that citizen engagement “[is] not working good enough and fast enough. (…) we try with the law to push that mechanism a little forward or to push it to accelerate. And the law isn't very important in the sense that it's introducing all kinds of new things. It's more sort of getting into law a lot of things which are already there and working” (Expert_7.110). The second aspect is addressing the low institutional trust of the public in the role of healthcare insurers in the Dutch healthcare system (Expert_10.185, Additional file 4).

Overall, experts did not expect the amendment to the Health Insurance Act to stand as a game-changer towards accelerating citizen engagement (Expert_4.47, Additional file 4); instead, they expected the legislation to mainly enhance how healthcare insurers document what they have already been doing towards operationalizing citizen engagement: “(…) we will focus on the way we report on that [citizen engagement]. Now, we do a ton of things, and we don't count it, or we don't make an annual report of that… The new legislation, maybe has the effect that things that we already do, we make that more transparent, (…) but the actual relevant instruments can still be zero” (Expert_4.49). Hence, it was noted that for healthcare insurers to further engage citizens, the focus should then be “(…) on getting the incentives right, rather than have all kinds of legal constructions to involve consumers (…)” (Expert_5.60).

During the six focus group meetings, we engaged with 29 representatives of healthcare insurers: 24 participants were employed at participating healthcare insurers, and five participants were insured people with seats at the council of insurees of each participating healthcare insurer. Healthcare insurers’ representatives recognized a changing context in the Dutch healthcare system, which supports an evolution from being a healthcare insurer towards becoming a health insurer. This evolution entails a greater focus on people-centred value-based principles, including broadening the services covered by healthcare insurers, such as preventive care. Healthcare insurers’ representatives identified societal and political momentum, rather than market forces, as instrumental in shaping this change. One participant emphasized that “It [evolving from a healthcare insurer to a health insurer] is actually essential. Otherwise, the system is not sustainable” (Focus_group_1.4). However, preparing for this evolution is not yet fully embedded in the corporate culture and strategy of some of the participating healthcare insurers. Nevertheless, all representatives of participating healthcare insurers agreed on the central role of citizen engagement for the evolution from healthcare insurer to health insurer to occur in Dutch society.

### The viewpoint of the healthcare insurers’ representatives on the operationalization of citizen engagement

Based on the data collected during the two phases of this study, we found that healthcare insurers are following four strategies towards operationalizing citizen engagement by: (1) broadening the *population health orientation* of the healthcare insurer; (2) *empowering the insured people* by developing and improving mechanisms whereby citizens can be engaged; (3) strengthening features of *data governance* at all levels for effective use of value-driven data; (4) implementing *financial and incentive mechanisms* among healthcare providers in support of value-based healthcare by offering and testing new approaches to healthcare procurement (Table 3). Both experts and representatives of healthcare insurers accounted for the context of a healthcare insurer and their corporate culture to shape the initiatives and extent to which healthcare insurers operationalize citizen engagement.

**Table 3:** Identified strategies and initiatives used by healthcare insurers to operationalize citizen engagement in the Dutch healthcare system

Strategy	Identified factors contributing to the success of citizen engagement initiatives	Illustrative cases
Population health orientation	• A well-established relationship with municipalities before the rollout of any initiative • Sufficient regional market share of the healthcare insurer • Broad support of stakeholders in the community (e.g. GPs) • The design of initiatives should be evidence-based	• Collaborating with municipalities on aiding people with financial problems • Convening focus groups of citizens for thematic discussions • Offering preventative programmes and e-health solutions promoting healthy lifestyles
Empowering the insured	• Moving towards a *professionalization* of the council of insurees • Diversify communication channels and use clear-cut messages to help inform citizens’ decision-making • Ability to connect and to communicate with citizens sharing similar needs, within the provisions of the law	• Engaging the council of insurees early in the healthcare procurement process • Informing insurees on provider benchmarking results in regular newsletters • Omnichannel support to insurees who need assistance regarding navigating the healthcare system
Data governance	• Data fitness for purpose and use • Data linkage between health information systems and actors at the national and regional level • Data availability on value for patients	• Measuring care providers’ performance towards supporting quality assurance and monitoring initiatives • Regional profiling of communities • Monitoring insurees’ care experiences and satisfaction by conducting complaints management
Financial and incentive mechanisms	• Sufficient regional market share of a healthcare insurer may lead to a representation model (i.e. a healthcare insurer negotiating with providers on behalf of other healthcare insurers) • Sharing of benefits among actors who invest in prevention and health promotion	• Incentivizing value-based healthcare by offering multiyear or volume-free contracts to selected providers • Testing new approaches to healthcare procurement (e.g. selective contracting)

#### Population health orientation

Healthcare insurers are broadening their population health orientation by partnering with other actors towards improving the health status and outcomes of communities. This broadening is facilitated by regional partnerships and coalitions of healthcare insurers, such as those with municipalities and citizen-led movements (Focus_group_5.181, Additional file 4). Another illustration is the broadening of healthcare insurers from bulk collective contracts with employers to working “together with the municipality, (…) targeting specifically people with financial problems, so that municipalities take initiatives to help these people” (Focus_group_2.51), particularly in exploring other ways of addressing preventive care and health promotion needs. From the healthcare insurers’ lenses, four features were identified as critical for the success of regional interventions: (1) a well-established relationship with the municipalities involved; (2) sufficient regional market share of the healthcare insurer; (3) broad support of the stakeholders in the community (e.g. general practitioners [GPs]); (4) robust anchoring in scientific evidence. However, key challenges are yet to be addressed to optimize such collaborations among actors in the healthcare system, particularly in terms of clarifying “(…) which parts could each stakeholder complement in collaboration and what is needed from each other, [considering that] it could vary between regions/municipalities. This is what makes it complex” (Focus_group_5.190).

#### Empowering the insured

All healthcare insurers have shown initiatives towards strengthening the voice of the insured in decision-making processes. For example, initiatives are pursued to engage local communities in decision-making, but these are still challenging to operationalize (Focus_group_3.101, Additional file 4). Healthcare insurers were at different stages of exploring the full potential of their council of insurees; some healthcare insurers had more mature models than others. The amendment to the Health Insurance Act was seen as an opportunity to explore different approaches to engaging the council of insurees in healthcare procurement (Focus_group_1.25, Additional file 4). Engaging the insured comes with its challenges, particularly in more complex topics such as healthcare procurement: “It is particularly challenging to give insurees sufficient baggage to be able to give input. It is so complex to understand the impact on healthcare. They need to have the right information” (Focus_group_6.296). Besides the challenge of having a layperson meaningfully understand the legal framework of healthcare procurement, “The involvement of clients is not always high. (…) the insurer needs to show better what is in it for clients when they are involved” (Focus_group_1.27). To overcome these hindrances, two solutions were signalled: first, moving towards a professionalization of the council of insurees; second, strengthening communication channels with the insured, with more straightforward messages that can better inform their decision-making.

#### Data governance

Healthcare insurers’ representatives stated that they had limited access to intelligence that is fit for purpose and supportive of healthcare insures' role as a value-based purchaser of care. Most of the data available to healthcare insurers are claims data; patient-reported outcome (PROMs) and experience (PREMs) measures are still very limited. The low availability of patient-reported data is linked to the trust levels of care providers and citizens in healthcare insurers’ uses of these data. For example, care providers are reluctant about the potential benefits of sharing these data in the context of the data protection legislation (Focus_group_5.216 and Focus_group_2.77, Additional file 4). During the focus groups, representatives of healthcare insurers mentioned that the data available on their end are used for three key purposes: (1) regional profiling of communities; (2) healthcare procurement; (3) measuring performance in support of quality assurance and monitoring initiatives (Focus_group_1.29, Additional file 4). Although healthcare insurers are limited in conducting individual-level profiling because of privacy laws, most healthcare insurers’ representatives stated that through collaborations with regional partners (e.g. municipal health services—GGD), they had been able to link data at the community level. This supports healthcare insurers in better understanding the profile and needs of communities and the use of intelligence in support of value-based healthcare procurement.

#### Financial and incentive mechanisms

The approaches for procuring care seem to be evolving with the increasing presence of healthcare insurers in regions. Suppose a healthcare insurer has sufficient market share in a region. In that case, this could lead to a representation model (i.e. where a leading healthcare insurer negotiates with care providers and municipalities on behalf of other healthcare insurers), facilitating opportunities for shared learning. Conversely, a limited market share in a region could have implications for a healthcare insurer’s willingness to invest in that region (Focus_group_3.93, Additional file 4). Healthcare insurers’ representatives advocated for a better alignment of financial and incentive mechanisms across actors in the healthcare system. Aligning financial and incentive mechanisms can create value and strengthen the economic sustainability of the health (care) system. For example, nudging value creation via care providers could be achieved via multiyear or volume-free contracts. However, healthcare insurers’ representatives signalled that a pooled budget could be the instrument with the most significant effect on steering change. A pooled budget could strengthen trust among actors and spur investments in prevention and health promotion. In parallel, a financial model should be in place to divide among healthcare insurers the (economic) benefits of investing in a population’s health.

## Discussion

With this study, we set out to explore the role of healthcare insurers in operationalizing citizen engagement in the Dutch healthcare system. The findings indicate that healthcare insurers are increasing their presence in regions and wish to broaden their role in health promotion and preventive care. Citizen engagement results from the evolving positioning of healthcare insurers in better understanding and meeting the needs, expectations and preferences of insurees and enhancing their end-to-end experience. The broader engagement of citizens by healthcare insurers requires a long-term culture change. It is shaped by healthcare insurers’ societal background anchored in solidarity values and forces of the regulated competitive market. Healthcare insurers have been developing several initiatives to engage citizens, the insured and patients in product and policy development cycles, including healthcare procurement. Healthcare insurers’ key strategies towards a more significant and sustainable citizen engagement included (1) broadening their population health orientation; (2) developing and improving mechanisms for engaging citizens, notably insured people; (3) strengthening features of data governance at all levels for effective use of value-driven data; (4) implementing financial and incentive mechanisms among healthcare providers in support of value-based healthcare by offering and testing new approaches to healthcare procurement. However, citizens’ low institutional trust in the role of healthcare insurers in the healthcare system undermines progress.

Successful implementation of value-based care across the health system requires citizens (including insured people and patients) to be engaged in health policy decision-making processes [4, 5, 18]. Healthcare insurers—despite the persistent public perception of being profit-driven and limiting one’s freedom of choice of care providers—have been elaborating initiatives supporting citizen engagement (e.g. the council of insurees as a governing body of the healthcare insurer) [20]. Yet, citizen engagement has not been implemented fully in product and policy development cycles, partly because of the corporate culture and organizational aspects of healthcare insurers and because of power imbalances in the healthcare system [21]. In addition, low institutional trust in healthcare insurers and their corporate identity may have led citizens to perceive themselves solely as consumers in the health insurance market rather than partners accountable for co-producing people-centred value-based healthcare [22–25]. Such shortcoming is expected to be addressed with the amendment of the Health Insurance Act, where the engagement of the insured by healthcare insurers is legally formalized. Time will tell whether this amendment reinstates among citizens the general perception of the foundational solidarity function of most Dutch healthcare insurers.

Healthcare insurers have been strengthening their regional orientation, with varying degrees of maturity and cooperation with other actors in the health system, such as competing healthcare insurers, municipalities, care providers and communities. This regional orientation is partially influenced by the historical background of healthcare insurers. It is also a means of operationalizing people-centred value-based care by better addressing communities’ care needs and expectations and enhancing end-to-end citizens’ experiences. Additionally, healthcare insurers could be better positioned for strengthening a trust-based relationship with citizens by being closer to communities. Yet, the lack of clarity about the role of health system actors in citizen engagement serves to perpetuate power imbalances in the system [26]. If healthcare insurers are to address such power imbalances and strengthen proximity to communities, institutional trust in healthcare insurers could be strengthened; in return, healthcare insurers could gain negotiating power with care providers, leading to effective implementation of procuring policies guided by people-centred value-based principles.

The current health information landscape is not sufficiently robust to support healthcare insurers in operationalizing care procurement policies anchored in people-centred value-based principles. Aside from the healthcare insurers’ poor availability of care quality and outcome data (notably, PROMs and PREMs) [12], unclear regulation about data custodianship hampers a greater use of intelligence towards supporting value creation. It is not clear which data each actor in the system should have access to and what the value chain of data could be like. Also, data protection and privacy regulations limit the ability to link data within and between actors [27]. However, healthcare insurers seem to maximize the use of data and analytics-driven approaches with available data to steer the development of new policies and products, particularly at the regional level. Fostering a culture of transparency and reciprocal benefits to sharing data are needed to align actors towards better addressing the needs and expectations of the insured [28].

### Strengths and limitations

This study was enriched by engaging representatives of the four largest healthcare insurer groups in the Netherlands, including varying views of experts and engaging representatives of the council of insurees of each participating healthcare insurer. Our findings are relevant to understanding how healthcare insurers are operationalizing citizen engagement before changes are introduced to their business model because of the amendment to the Health Insurance Act (*wet verzekerdeninvloed*). Also, this study helps in understanding tensions between the healthcare insurers’ historical background and their positioning and functioning in a regulated competitive market. The entire research team was involved in data collection to strengthen the study’s validity, and two researchers (ÓBF and VB) led the data analysis. Reliability was strengthened with cascading rounds of data collection, comprehensive data use and continuous data analysis. Generalizing the findings to other countries with insurance-based healthcare systems, including those in low- and middle-income countries according to the World Bank’s classification, should be done with caution. For example, contextual factors, such as the organization and digitalization of the healthcare system, the broader policy environment and power distribution among actors in the system, and the extent to which these affect applications of citizen engagement by healthcare insurers, are heterogeneous. Yet, this study can help inform other countries with insurance-based healthcare systems in steering their healthcare insurers towards inclusive governance approaches by strengthening citizens’ engagement in health policy decision-making. This is particularly relevant in contexts of limited resources and increasing health expenditure, where the citizens’ voice can play an active role in defining priorities and steering healthcare insurers’ healthcare procurement policies towards people-centred value-based values. A limitation of the study relates to having selected only the four largest health insurance companies (with 81% of the market share); we acknowledge that healthcare insurers with lower market share could have generated different narratives and contributed to maximal theoretical representation. Finally, another limitation is the limited opportunity for citizens to exercise their voice and be involved in this research. Although all study participants are themselves citizens, and we ensured the participation of representatives of the council of insurees of the participating healthcare insurers, pursuing other meaningful forms of involving the broader public offers an opportunity to better understand how citizens perceive being engaged by healthcare insurers in healthcare procurement decision-making processes.

## Conclusions

This study explored the role of healthcare insurers in operationalizing citizen engagement in healthcare procurement, notably how the rollout of an amendment to the Health Insurance Act is expected to change healthcare insurers’ practices of engaging citizens in procurement decision-making processes. Dutch healthcare insurers seem to be strengthening the community orientation of their functioning. The focus on regions has led healthcare insurers to change their relations with citizens in their various roles (as insured or patients) and patients’ representatives. In general, healthcare insurers already had in place many initiatives supporting citizen engagement. The immediate practical effect of the amendment to the Health Insurance Act will likely be twofold: broadening the role of the council of insurees in decision-making processes, and systematically documenting the efforts set forth by healthcare insurers in engaging citizens. Also, other financial mechanisms are deemed necessary to streamline value creation across actors, such as a pooled budget. Further research is needed to better understand how the regulated competitive market could be hampering the engagement of citizens in healthcare procurement decision-making and value creation from the citizens’ perspective.

## Supplementary Information

**Additional file 1.** Template for semi-structured interviews with academics and policy experts.

**Additional file 2.** Invitation letter.

**Additional file 3.** Discussion statements to be debated with healthcare insurers’ representatives.

**Additional file 4.** Supporting quotes from experts and healthcare insurers’ representatives.

## Data Availability

Data sharing is not applicable to this article as no datasets were generated or analysed during the current study.

## Abbreviations

PROMs: Patient-reported outcome measures
PREMs: Patient-reported experience measures
GPs: General practitioners
