# Supplementary material for: Citizen engagement in healthcare procurement decision-making by healthcare insurers: recent experiences in the Netherlands
Source: Health Res Policy Syst. 2022 Dec 22;20:137. doi: 10.1186/s12961-022-00939-7 (PMC9773595; doi:10.1186/s12961-022-00939-7)
Supplement: Supplementary file 1 — Additional file 1. Template for semi-structured interviews with academics and policy experts. [file 12961_2022_939_MOESM1_ESM.pdf]

Additional file 1

Template for semi-structured interviews with academics and policy experts

INTERVIEW SCRIPT
EXPERTS

Consent

1. Notes will be made during the interview to assist the researcher in accurately recalling and capturing your insights.
2. All retrieved data will be only analysed by the research team and limited to this use.
3. These data may be used in academic papers, policy papers, or in other media that the research team may produce, such as spoken presentations.
4. Individual participants' names will remain anonymous.
5. Agree to have the interview audiotaped?

QUESTIONS

1. **WHY are health insurers pushing consumer engagement?**

    a. WHY is the government pushing forward new legislation on consumer engagement in healthcare purchasing by insurers?

2. **HOW have health insurers organized consumer engagement thus far?**

    a. How will health insurers adapt if a new version of the Health Insurance reform act comes into force?

3. **WHAT practices and tools do health insurers have in place to collect, analyse, and report on person-reported data?**

    a. WHAT is known about the effects of using those kinds of data?
    b. WHAT facilitating and hindering factors influence the use of these data?
