# Supplementary material for: Citizen engagement in healthcare procurement decision-making by healthcare insurers: recent experiences in the Netherlands
Source: Health Res Policy Syst. 2022 Dec 22;20:137. doi: 10.1186/s12961-022-00939-7 (PMC9773595; doi:10.1186/s12961-022-00939-7)
Supplement: Supplementary file 2 — Additional file 2. Invitation letter. [file 12961_2022_939_MOESM2_ESM.pdf]

Additional file 2

Invitation letter

Invitation letter

to take part in a virtual focus group meeting on consumer engagement

1. Context

HealthPros is a Marie Skłodowska-Curie Innovative Training Network for Healthcare Performance Intelligence Professionals (www.healthpros-h2020.eu). The network provides project-based training for 14 PhD candidates (HealthPros fellows) into a first generation of Healthcare Performance Intelligence Professionals that will be able to systematically link health data and strategic planning decisions.

One of the fellows [name of the Fellow] is working on a project on the use of consumer-data in the Dutch health insurance market, focusing on consumer involvement in health care purchasing by Dutch insurers. The fellow is originally based at [Fellow's affiliation] and is supported by principal investigators [Principal Investigator's name] and [Principal Investigator's name] from [Principal Investigators' affiliation].

2. Why is your participation important?

We are inviting you to participate because of your expertise and viewpoint on consumer engagement, which gives you a privileged outlook on the central topic of discussion at the focus group meeting. The focus group will provide an opportunity for you to know about key findings of a recently completed study that aimed to better understand the why, what, and how health insurers use patient-reported data.

3. Output of your participation

Your input is important to us. All focus group participants will receive a summary document with key messages that emerged at the event. This document and your reaction to it, if any, will be taken into consideration together with other evidence to produce a scientific publication.

4. Confidentiality

Your personal details will be handled with strict confidentiality. The discussions from this event will be summarized anonymously. Your personal details (name, role and contact information) will be used by the organizers only for coordination purposes (e.g., send you a summary of the focus group discussions). You will be asked to provide your consent prior the beginning of the event to confirm your understanding and agreement to participate.

5. Location and time commitment

The focus group will be held **virtually** on a date that will soon be disclosed.

6. Language

The event will be held in English and Dutch. A round of introductions and a short presentation about the topic of discussion will be conducted in English. Participants may choose to express themselves both in Dutch and English.

7. Focal point at [health care insurer] for information

The focal point at [health care insurer] is [focal point name] [focal point email].

8. Research team contact information

The virtual focus group will be moderated by: [Study team]. To contact the focus group moderators directly, please write to [Fellow's email address].
