# Supplementary material for: Citizen engagement in healthcare procurement decision-making by healthcare insurers: recent experiences in the Netherlands
Source: Health Res Policy Syst. 2022 Dec 22;20:137. doi: 10.1186/s12961-022-00939-7 (PMC9773595; doi:10.1186/s12961-022-00939-7)
Supplement: Supplementary file 3 — Additional file 3. Discussion statements to be debated with healthcare insurers’ representatives. [file 12961_2022_939_MOESM3_ESM.pdf]

Additional file 3

Discussion statements to be debated with health care insurers' representatives

English	Dutch
From health care insurers towards health insurers	Het is wenselijk en mogelijk dat zorgverzekeraars in Nederland gezondheidsverzekeraars worden
The new law will help to increase the involvement of communities with their care system	De nieuwe wet over inspraak van verzekerden kan helpen de betrokkenheid van een gemeenschap bij de zorg te vergroten
The data insurers have about the insured are insufficient for purchasing quality of care	Op dit moment zijn de gegevens die de verzekeraar heeft over verzekerden onvoldoende om gericht zorg op kwaliteit in te kopen
Cost control and strengthened value creation in health care is only feasible through increased involvement of citizens/patients	Kostenbeheersing en versterkte waardecreatie in de zorg zijn alleen mogelijk door een versterkte betrokkenheid van burgers/patienten
