# Supplementary material for: Citizen engagement in healthcare procurement decision-making by healthcare insurers: recent experiences in the Netherlands
Source: Health Res Policy Syst. 2022 Dec 22;20:137. doi: 10.1186/s12961-022-00939-7 (PMC9773595; doi:10.1186/s12961-022-00939-7)
Supplement: Supplementary file 4 — Additional file 4. Supporting quotes from experts and healthcare insurers’ representatives. [file 12961_2022_939_MOESM4_ESM.pdf]

Additional file 4

Supporting quotes from experts and health care insurers' representatives

Section in the manuscript	Coding	Quote
**3.1. The role of health care insurers in citizen engagement in the Dutch health care system**	Expert_6.70	(…) we say [that] their role [health care insurers] is to critically purchase, maybe for their defined population be the critical purchaser. So, it [care delivery] must be efficient and must be of good quality (...) But I believe [that] in the Dutch society, there's a mismatch what we have given that role, that they are doing that for us (...) And the providers are just… (…) this relationship here is way stronger [referring to the relationship between care providers and citizens], if providers are just going to scream [because health care insurers] (…) are negotiating too hard, they [citizens] believe them, and there's a mistrust to them [health care insurers]. So, we don't trust… hum... there's a mismatch between what we perceive as society what they [health care insurers] should do and what's in the law, what the tasks are. And that's exactly what's fundamentally… what I think is a huge problem in the Netherlands.
	Expert_10.185	(...) they're [policymakers] trying to build trust in insurance companies, because what we've seen in the last decade is that we have a system based on regulated markets, in which there is a very important role for insurance companies to purchase good quality of care at preferably low cost for their insured population. But every time they try to do that, they encounter... hum... bad press that damages their reputation and people question their sincerity and actually, [they are] trying to... to promote quality of care, (…) [but] the public doesn't have a lot of trust in insurance companies. But by emphasizing consumer engagement in the things that insurance companies do, then the Government may hope to revitalize the trust in them and in their behaviour.
	Expert_4.47	(…) doesn't get you to the to the kinds of engagements you really want, because the engagement you really want is in the level of redesigning health care processes, together with providers; and then transform it, to make it applicable for all patients, for all members. And this is a much more rapid prototyping design and sort of design-thinking on how to use the engagement of patients and members.
**3.2.1. Population health orientation**	Focus_group_5.181	(...) we collaborate with two municipalities in preventing health issues of vulnerable people in those municipalities. We also collaborate with patient organisations by asking what the needs of specific patient groups are and understand what kind of interventions are needed, including prevention. One hinderance is on understanding 'What does prevention include?' and 'Who to collaborate with?' because if this is not done in collaboration with municipalities, the insurer needs to pay for it.

**3.2.2. Empowering the insured**	Focus_group_3.101	(...) [in some regions, the health care insurers] have a clear structure to work with, in other regions there is a lack of a clear structure to involve local communities. (...) we try, when relevant, to involve the client board [*leden raad*]. We ask about patient experiences in care provision. Also, about shared development… We need to make the shift from what is the matter with you to what matters to you.
	Focus_group_1.25	We have a client board [*leden raad*] that in past would be engaged at the end of purchasing policy cycles. Now, we want to engage the board earlier in the development process of new purchasing policy as representative of the insurees. In the future, there will be two moments of interaction: the first for drafting early ideas and another at a later stage, toward the pre-final phase, for validating with the representatives of the insured. The law also states that insurees should have a say about the communication policy and the insurer purchasing. This is a different route, and we want to offer this opportunity. We are now in the process of finalizing this and publishing on our website. Insurees engagement is voluntary, and we wisht to develop a database of insurees that we could consult on specific topics or challenges.
**3.3.3. Data governance**	Focus_group_5.216	(…) I would say that we have to distinguish between *having the data* and *being able to use those data*; these are different aspects. (...) Sometimes we have PROMs and PREMs data, but one question is if it really represents what patients' value most. (…) We are always searching for room to use those data. (…) But in general, people also fear privacy violations if the insurers get too much power.
	Focus_group_2.77	(...) Providers are fearful of how we are using this information, and thus, it should be clearer that we, as insurers, plan to use these data for purchasing care. Also, these data need to be sufficiently distinctive, i.e. show real and meaningful differences between the quality of providers.
	Focus_group_1.29	[We use data] For value-based health care contracts for hospital care mostly, for which we try to integrate the whole patient journey. This trajectory started years ago, but there is lack of PROMs. There are process data, but we need outcome data. There are few PROMs available and information about treatments are lacking. We cannot collect PROMs ourselves; it does not fit our role. Notwithstanding, we try to influence certain specialists (e.g., breast cancer, cataracts, knee) to start collecting those data. But this is time consuming and labour intensive. So, developing value-based health care is difficult, although we do like this strategy for health care purchasing.
**3.3.4. Financial and incentive mechanisms**	Focus_group_3.93	(…) some parties don't want to invest because they won't see the profits [of investments in prevention or health], despite the long-term benefits. (...) We only choose those regions where we are market leader because we have sufficient volume to undertake such investments, and to collaborate with other insurers.
